# Retraction Note: N^6^ -methyladenosine modification of circMARK2 enhances cytoplasmic export and stabilizes LIN28B, contributing to the progression of Wilms tumor

**DOI:** 10.1186/s13046-025-03326-6

**Published:** 2025-02-18

**Authors:** Guannan Shu, Zhang Zhao, Tianxin Zhao, Changmi Deng, Jiangquan Zhu, Yufeng Han, Minyu Chen, Jiajia Jing, Gaochen Bai, Dian Li, Feng Li, Jing He, Wen Fu, Guochang Liu

**Affiliations:** 1 Department of Urology, Guangzhou Women and Children’s Medical Center, Guangzhou Medical University, Guangzhou Institute of Pediatrics, Guangdong Provincial Clinical Research Center for Child Health, Guangzhou, 510623 Guangdong China; 2 Department of Pediatric Surgery, Guangzhou Women and Children’s Medical Center, Guangzhou Medical University, Guangzhou Institute of Pediatrics, Guangdong Provincial Key Laboratory of Research in Structural Birth Defect Disease, Guangdong Provincial Clinical Research Center for Child Health, Guangzhou, 510623 Guangdong China; 3 Department of Urology, The First Afliated Hospital of Sun Yat-Sen University, Guangzhou, 510080 Guangdong China; 4 The First Afliated Hospital, Sun Yat-Sen University, Guangzhou, 510080 China; 5 State Key Laboratory of Virology, Center for Biosafety Mega-Science, Wuhan Institute of Virology, Chinese Academy of Sciences, Wuhan, 430071 China

**Retraction Note** **:** **J Exp Clin Cancer Res 43, 191 (2024)**

**https://doi.org/10.1186/s13046-024-03113-9**

The authors have retracted this article. The article reports research that intended to use two lines derived from human Wilms tumour cells: G401 and SK-NEP-1. After publication, the authors were informed that these lines were in fact derived from a rhabdoid tumour of the kidney and a Ewing sarcoma, respectively. The authors have been offered an opportunity to submit an amended manuscript containing experiments using verified Wilms tumour cell lines, which will be sent for peer review.

All authors agree with this retraction.

